# Acute myeloid leukemia with unreported translocation (x; 3) (q24; p13): A case report

**DOI:** 10.1002/ccr3.8543

**Published:** 2024-02-20

**Authors:** Nasrin Gholami, Amirreza Khalaji, Maghsood Mehri, Saba Mehrtabar, Elham Habibzade

**Affiliations:** ^1^ Hematology and Oncology Research Center Tabriz University of Medical Sciences Tabriz Iran; ^2^ Connective Tissue Diseases Research Center Tabriz University of Medical Sciences Tabriz Iran; ^3^ Immunology Research Center Tabriz University of Medical Sciences Tabriz Iran; ^4^ Department of Medical Genetics Tabriz University of Medical Sciences Tabriz Iran; ^5^ Student Research Committee Tabriz University of Medical Sciences Tabriz Iran; ^6^ Faculty of Medicine Tabriz University of Medical Sciences Tabriz Iran

**Keywords:** acute myeloid leukemia, cancer, chemotherapy, translocation

## Abstract

**Key Clinical Message:**

Novel and rare chromosomal aberrations in AML are important to understand, particularly if associated with tumorigenesis and how they contribute to prognostic risk. It is important that acute leukemia be treated right away. Herein, novel (x; 3) (q24; p13) is described.

**Abstract:**

Acute myeloid leukemia (AML) is a cancer of the blood and bone marrow. It is the most common type of acute leukemia in adults. This type of cancer usually gets worse quickly if it is not treated. Here, we report an unusual case of AML with an unreported translocation associated with AML.

## 1 INTRODUCTION

Acute myeloid leukemia (AML) is typified by the neoplastic clonal expansion of precursor cells and the cessation of differentiation in the bone marrow.^1^ According to the American Cancer Society's projections for 2018, there were an estimated 19,520 new cases of AML and 10,670 deaths resulting from this disease in the United States. Additionally, it is estimated that approximately 0.5% of the US population will develop AML.^2^ The age‐adjusted mortality rates and incidence are reported as 2.8 and 4.3 per 100,000 individuals, respectively. Additionally, the 5‐year survival rate is noted to be 27.4%.^3^

The dysregulation of epigenetics plays a key role in developing various diseases, including cancer. The deconvolution of 200 AML genomes, led by The Cancer Genome Atlas, has demonstrated that a considerable number of these AMLs exhibit mutations in epigenetic regulators.^4^ Here, we now report an unusual case of AML with an unreported translocation associated with AML.

## 2 CASE PRESENTATION

A 28‐year‐old non‐smoker man initially presented with severe fatigue for a few weeks. He had been diagnosed with mild anemia, but no therapy was given. He also reported weakness and nighttime headaches. For any malignancies, his family history was unremarkable and his past medical history was negative. During the examination, the patient's vital signs were found to be within the expected range of values. There was no observation of lymphadenopathy or splenomegaly. Lung and heart examinations were unremarkable. The initial complete blood count revealed a total leukocyte count of 4530/mm^3^ (48% blasts, 53.6% neutrophils, 24.4% lymphocytes), hemoglobin of 8.6 g/dL, and a platelet count of 60,000/mm^3^ (Table 1). He was admitted to the hemato‐oncology ward due to his Bi‐cytopenia.

**TABLE 1:** The initial, first day of induction and last day of induction complete blood count.

CBC	Initial presentation	First day of induction	Last day of induction
White blood cell count	4530	1530	330
Neutrophils	2420	310	140
Lymphocytes	1100	710	180
Monocytes	370	410	10
Eosinophils	20	0	0
Basophils	20	10	0
Large unstained cells	590	90	0
Hemoglobin	8.6	8	7
Hematocrit	27.5	27.0	22.6
Platelets count	60,000	81,000	46,000
Red blood cell	3,480,000	3,350,000	2,850,000

**FIGURE 1:**
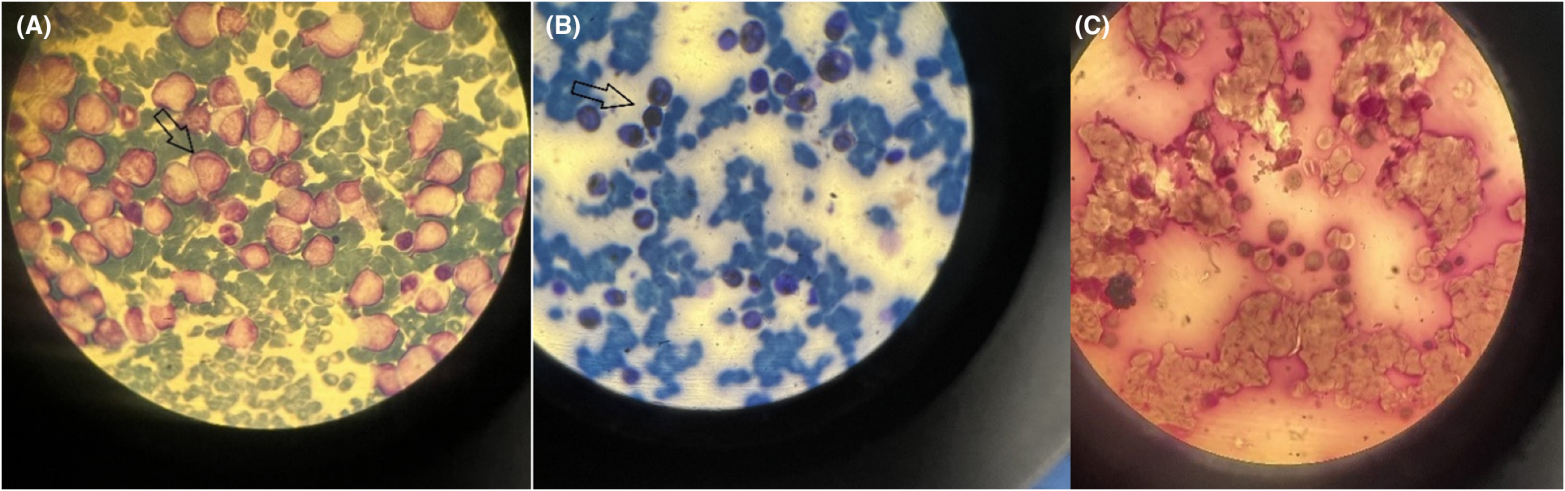
Bone marrow aspiration and biopsy. (A) Wright's Giemsa stain: show several myeloblasts with high nuclear to cytoplasmic ratio and homogeneous chromatin. (B) Sudan staining (heavily positive): A proportion of the blast cells strongly SBB‐positive, gives blackish brown discoloration to myeloid series cells. (C) Periodic acid‐Schiff (PAS) stain.

His peripheral blood smear showed 5000/mm^3^ white blood cell counts with 50% polymorph nuclear leukocytes (PMNs), 20% immature cells, 22% lymphocytes, and 8% monocytes with a platelet count of 60,000/mm^3^ (Table 2). Red blood cells were hypochromic microcytic with teardrops and anisocytosis. A diagnostic bone marrow aspiration and biopsy was performed (Figure 1). It should also be noted that evaluating essential gene mutations such as FLT3 and NPM1c in our center was impossible. His second PBS showed WBC: 10,000/mm^3^ with PMN 46%, lymph18%, mono:8%, and blast:28%. Samples of bone marrow were taken for immunophenotypic, morphologic, and genetic analysis. His bone marrow aspiration and biopsy were reported as diluted without particles, but about 80% of cells were seen in filtered blasts.

**TABLE 2:** Patient's peripheral blood smear.

Peripheral blood	Before bone marrow biopsy	Simultaneous with bone marrow biopsy
White blood cell count	5000	10,000
Immature cell	20%	28%
Polymorphonuclear neutrophils	50%	46%
Lymphocytes	22%	18%
Monocyte	8%	8%
Platelets count	60,000	60,000

**FIGURE 2:**
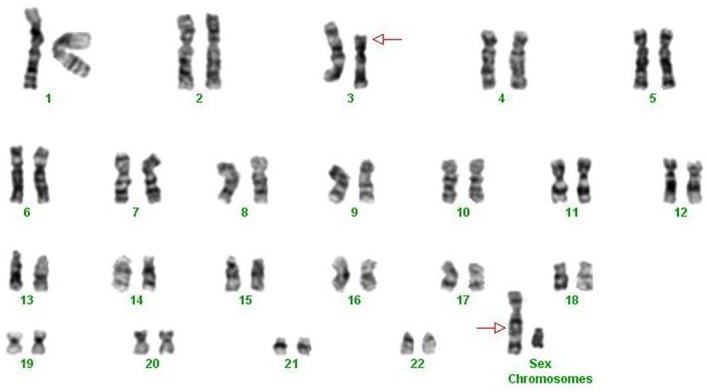
Patient's karyotype that shows aberrant male chromosomal complement, with the only aberration being a translocation between the short arm of chromosome 3 and the long arm of chromosome X marked in the arrow.

In the cells analyzed, cytogenetic examination revealed an aberrant male chromosomal complement, with the only aberration being a translocation between the short arm of chromosome 3 and the long arm of chromosome X (Figure 2). This translocation is a brand‐new anomaly in hematological malignancies that has not been documented in any of the references (such as the Mitelman database). At first, we suspected a congenital anomaly, but after routinely culturing mitogen‐stimulated peripheral blood cells, we found that the abnormality was disease‐related and absent in normal cells. In his flow cytometry, CD13, CD33, CD45, CD117, and HLA‐DR were 50%, 61%, 83%, 33%, and 46% reported, respectively (Table 3). The flow cytometric analysis and morphology revealed that the case was diagnosed with AML.

**TABLE 3:** Patient's flow cytometry.

Marker	Percentage	Marker	Percentage
CD19	0	CD10	0
CD22	0	CD11b	0
CD2	0	CD45	83
CD3	0	CD41	0
CD 7	0	CD34	0
CD13	50	CD38	66
CD15	0	CD64	5
CD14	5	CD117	33
CD33	61	HLA‐DR	46
		GlycoA	0

Induction chemotherapy was started for the patient with 7 + 3 (cytarabine 100 mg/m^2^/day continuous IV infusions for 7 days and danorubicinb 12 mg/m^2^/day for 3 days). On his first and last induction days, CBC was reported in Table 1. After the end of the chemotherapy induction, the patient was discharged from the hospital in a wealth condition. The patient is also under the supervision of our center doctors for further treatment and follow‐ups.

## 3 DISCUSSION

According to our knowledge, this is the first patient with AML with translocation (x; 3) (q24; p13) to be described. Genetic analysis is essential in classifying AML.^5^, ^6^, ^7^ Cytogenetic abnormalities are common in 50% of patients with AML. Chromosome abnormalities are used to classify patients with AML, regardless of blast count. The cytogenetic analysis also reveals prognostic and therapeutic consequences and can help evaluate the response to therapy, and will likely play a significant part in abnormality‐tailored treatments in the future. Likewise, mutational analysis is particularly vital in diagnosing and treating AML.^5^

Three distinct regions on the short arm of chromosome 3 have been associated with tumorigenesis. One of the previously mentioned regions is situated at the chromosomal locus 3p13 ~ p14.2.^8^ Historically, this region has been identified as a common human chromosomal problematic locus.^9^

It is hypothesized that the human chromosomal region 3p12‐p23 contains at least three tumor suppressor genes associated with lung cancer, renal cell carcinoma (RCC), and other neoplasms.^10^, ^11^

A study was conducted in 2007 to examine the cytogenetic alterations in 38 instances of renal tumors in correlation with the histopathological observations. The present study reveals that the structural rearrangements observed in the 3p region were exclusive to clear cell renal cell carcinoma (RCC). Notably, the translocation event between chromosomes 3p13 and 5q22 was the most frequently observed structural rearrangement in this context.^8^ The early stages of tumorigenesis are characterized by deletions occurring at the short arm of chromosome 3.^8^

Another study has demonstrated that the elimination of 3p13 characterizes a unique and forceful molecular subgroup of ERG‐positive prostate cancers, conceivably instigated by the deactivation of numerous tumor suppressors.^12^

The gene FOXP1 displays broad expression across various adult tissues; however, neoplastic cells frequently manifest a significant alteration in the localization or level of FOXP1 expression. The genomic locus of the human FOXP1 gene is situated on chromosome 3p13.

Forkhead box P1 is a transcription factor that regulates tissue and cell‐type‐specific gene transcription during development and adulthood. It has potential tumor suppressor properties and is located within a tumor suppressor region.^13^

The involvement of FOXP1 has been observed in various physiological contexts, such as the development of B‐cells, the differentiation of monocytes, and the regeneration of lung epithelia. The gene in question exhibits dual functionality in cancer, serving as both an oncogene in B‐cell lymphoma, ovarian cancer, and hepatocellular carcinoma and a tumor suppressor in T‐cell lymphoma, NSCLC (Non‐Small Cell Lung Cancer), and colorectal cancer.^14^, ^15^, ^16^, ^17^, ^18^, ^19^, ^20^, ^21^

Additionally, there exists supporting evidence indicating that the expression of FOXP1 in cells affected by breast cancer helps reduce the production of cytokines that attract T‐cells, thereby preventing the infiltration of stated cells.^14^, ^22^

FOXP1's function in T‐cells may lead to T‐cell lymphoma.^14^, ^22^

Forty‐five percent of patients (*N* = 5 of 11) in the study of Milosevic et al. carried deletions mapping to the transcription factor FOXP1.^23^

Previous studies have demonstrated that specific translocations in AML can predict response to therapy and overall survival. For example, patients with AML and the t(15;17) translocation, which results in the PML‐RARA fusion gene, have a better response to all‐trans retinoic acid (ATRA) therapy and a higher overall survival rate compared to those without this translocation.^24^, ^25^ Similarly, patients with AML and the t(8;21) translocation have a more favorable prognosis than patients without this translocation.^26^, ^27^

Seipel et al. at 2020 concluded that in AML patients receiving aggressive induction chemotherapy and autologous stem cell transplant, FOXP1 predicts survival. Patients with high FOXP1 gene expression had shorter progression‐free and overall survival.^28^

Levavasseur et al. have shown that cytogenetically normal AML patients with high FOXP1 expression had worse survival. FOXP1 knockdown increased superoxide anion levels, oxidizing cells, and increased cellular oxidative stress.^29^

In 2023, Tang et al. identified 17 AML patients with a pericentric inv^3^ leading to MECOM rearrangement, one of them had breakpoints at 3p13 on 3p and 3q26.2 on 3q.^30^

Translocation (x; 3) (q24; p13) may cause chemotherapy refractoriness in AML. Unidentified companion gene mutations may also cause refractory illness. Collecting and reporting uncommon chromosomal abnormalities may help explain AML's pathophysiology and prognosis. Early identification of the disease during the clinical course may lead to better patient outcomes and management in the future. This may facilitate the selection of patients for more aggressive chemotherapy regimens and allogeneic stem cell transplants.

## 4 CONCLUSION

This case demonstrates that translocation (x; 3) (q24; p13) could result in AML. According to the limitations of this article, it was not possible to perform NGS and molecular pattern analysis on AML; therefore, it is recommended that further research be conducted on this topic in future research.

## AUTHOR CONTRIBUTIONS

**Nasrin Gholami:** Supervision. **Amirreza Khalaji:** Data curation; writing – original draft. **Maghsood Mehri:** Data curation. **Saba Mehrtabar:** Writing – original draft; writing – review and editing. **Elham Habibzadeh:** Data curation.

## FUNDING INFORMATION

This research did not receive any specific grant from funding agencies in the public, commercial, or not‐for‐profit sectors.

## CONFLICT OF INTEREST STATEMENT

None.

## ETHICS STATEMENT

This study was performed according to the principles outlined by the World Medical Association's Declaration of Helsinki on experimentation involving human subjects, as revised in 2000, and has been approved by the ethics committee of the Tabriz University of Medical Sciences.

## CONSENT

Written informed consent was obtained from the patient to publish this report and clinical images. Consent has been signed and collected in accordance with the journal's patient consent policy.

## Data Availability

The data supporting the findings of this research are available upon reasonable request from the corresponding author.
